# Altered body composition in children and adolescents with achondroplasia

**DOI:** 10.3389/fnut.2026.1895101

**Published:** 2026-07-22

**Authors:** Javier Gómez-Ambrosi, Isabel María Vegas-Aguilar, María del Mar Amaya-Campos, Patricia Yárnoz-Esquíroz, Rocío Fernandez-Jiménez, María García-Olivares, Camilo Silva, José Manuel García-Almeida, Gema Frühbeck

**Affiliations:** 1 Metabolic Research Laboratory, Clínica Universidad de Navarra, Pamplona, Spain; 2 CIBER Fisiopatología de la Obesidad y Nutrición (CIBEROBN), Instituto de Salud Carlos III, Pamplona, Spain; 3 Obesity and Adipobiology Group, Instituto de Investigación Sanitaria de Navarra (IdiSNA), Pamplona, Spain; 4 Institute for Nutrition and Health, University of Navarra, Pamplona, Spain; 5 Department of Endocrinology and Nutrition, Virgen de la Victoria Hospital, Malaga, Spain; 6 Instituto de Investigación Biomédica de Málaga (IBIMA)-Plataforma BIONAND, Malaga, Spain; 7 Department of Endocrinology and Nutrition, Clínica Universidad de Navarra, Pamplona, Spain; 8 Department of Endocrinology and Nutrition, Quironsalud Málaga Hospital, Malaga, Spain; 9 Faculty of Medicine, University of Malaga, Malaga, Spain

**Keywords:** achondroplasia, adolescent, air displacement plethysmography, body composition, children, sex

## Abstract

**Introduction:**

Achondroplasia is characterized by disproportionate short stature, yet little is known about how growth patterns and body composition differ from peers during childhood and adolescence. We aimed to compare anthropometric variables and detailed body composition profiles between children and adolescents with achondroplasia and age-matched controls.

**Methods:**

In this cross-sectional study, anthropometry and body composition were assessed in participants aged 6–18 years using air displacement plethysmography (BOD POD). Height, weight, BMI, fat mass (FM), fat mass index (FMI), fat-free mass (FFM), and fat-free mass index (FFMI), were compared between individuals with achondroplasia and controls.

**Results:**

Participants with achondroplasia showed significantly lower height and weight but higher BMI compared with controls (*p* < 0.05). FM (kg) was markedly lower in achondroplasia (*p* < 0.001), whereas FFM (%) and FFMI were significantly higher. In the overall sample and in females, the proportion of FFM was greater in achondroplasia (*p* < 0.05), whereas differences in males were not significant. Across all age strata, older age groups exhibited higher FM and FFM values, with consistent differences between groups for both components (main effect of group *p* < 0.01). Age correlated positively with FM and FFM in both groups, but correlations were stronger for FFM (controls: *r* = 0.75; achondroplasia: *r* = 0.87; both *p* < 0.001).

**Discussion:**

Children and adolescents with achondroplasia present a distinct body composition profile characterized by lower absolute fat and lean mass but proportionally higher FFM than controls. These findings highlight unique growth and body composition trajectories in achondroplasia and reinforce the importance of condition-specific reference values for nutritional and clinical assessment.

## Introduction

Achondroplasia is the most common type of skeletal dysplasia and the leading genetic cause of disproportionate short stature, with an estimated prevalence of 1 in 20,000 live births (1, 2). It results from a gain-of-function mutation the fibroblast growth factor receptor 3 (FGFR3) gene, leading to constitutive receptor activation and inhibition of endochondral ossification, particularly at the growth plate of long bones (3, 4). Affected individuals present with rhizomelic limb shortening, macrocephaly, and characteristic craniofacial and spinal features, while intelligence and life expectancy are typically unaffected (1, 2). Achondroplasia is a congenital skeletal dysplasia that is usually recognizable at birth due to its characteristic clinical features. Consequently, growth patterns diverge from those of average-stature children from early childhood onwards, affecting body proportions and potentially influencing body composition development throughout childhood and adolescence. Although classically regarded as a disorder of linear growth, achondroplasia has multisystemic implications that extend beyond stature, including orthopedic, neurological, and metabolic complications that often emerge during childhood and adolescence (5).

Anthropometric evaluation is paramount for both the diagnosis, as well as for tracking growth and evaluation of the response to treatments of children and adolescents with achondroplasia (6). The use of conventional anthropometric indices, particularly the body mass index (BMI), has been shown to misclassify people of normal stature of any age (7–9). In addition, because BMI is calculated from weight divided by height squared, it systematically overestimates adiposity in people with short stature, as height exerts a disproportionate effect on the denominator (6, 10). During these dynamic developmental stages, body composition undergoes substantial remodeling driven by hormonal, nutritional, and behavioral changes. In children with achondroplasia, disproportionate limb shortening and relative trunk enlargement modify the geometry and mechanical loading of the body, potentially influencing tissue distribution (6). Reduced mobility and lower levels of habitual physical activity have also been reported (11), which may further affect fat and lean mass accrual over time. However, the extent to which these structural and behavioral factors alter the proportional distribution of fat and lean compartments remains incompletely understood.

Accurate characterization of body composition in achondroplasia is, therefore, essential for both clinical management and research. From a clinical perspective, understanding the relationship between BMI, body fat percentage (BF%), and fat-free mass (FFM) provides a foundation for rational nutritional guidance, growth monitoring, and metabolic screening in pediatric populations. Establishing patterns of body composition in children and adolescents with achondroplasia appears critical also for evaluating both the safety and physiological effects of treatments.

Despite these important clinical and scientific considerations, evidence regarding body composition in children and adolescents with achondroplasia remains limited. Only a few studies have used precise measurement methods in sufficiently large populations or included direct comparisons with age and sex-matched typically developing controls (11–14).

The aim of this study was therefore to compare body composition profiles between children and adolescents with achondroplasia and age and sex-matched typically developing controls, using detailed measures of BF% and FFM obtained from air displacement plethysmography (ADP).

## Methods

### Study design and participants

**Figure 1:**
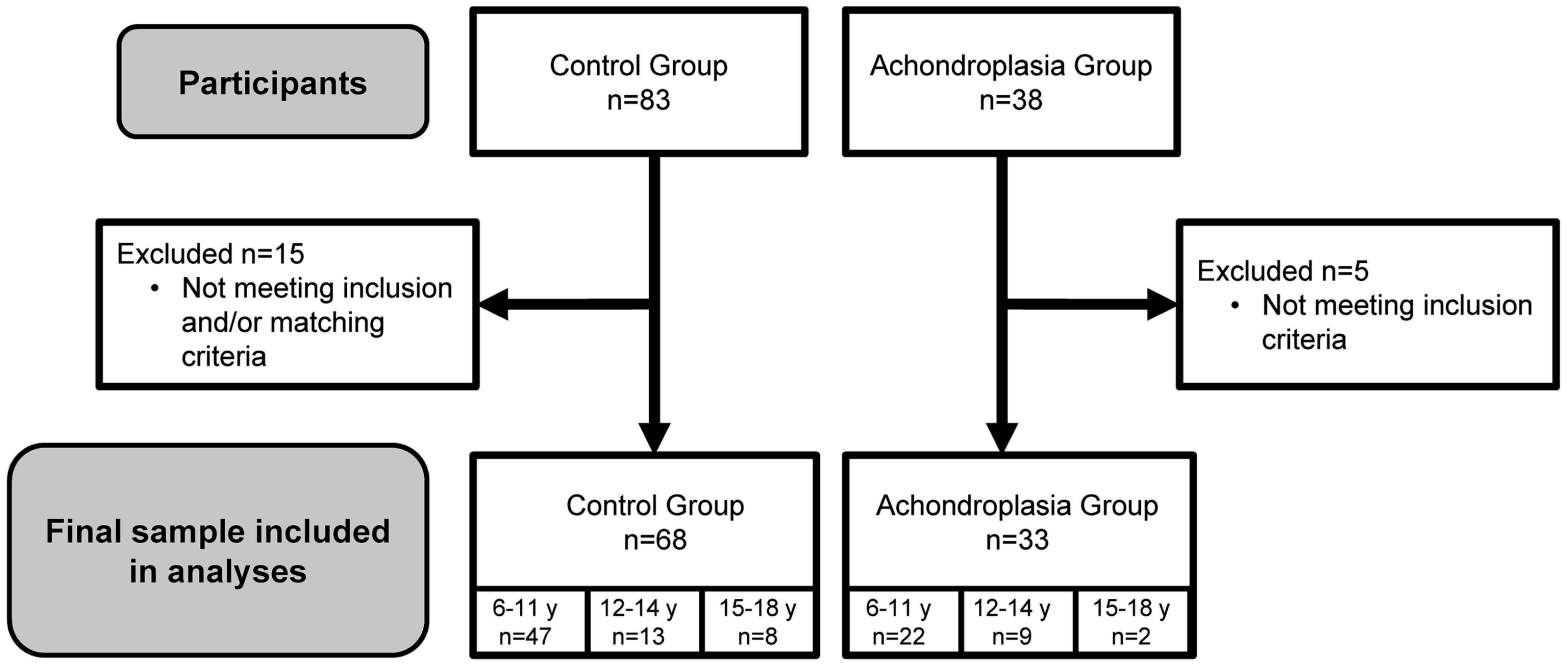
Flow diagram of participant recruitment and inclusion in the study. A total of 83 controls and 38 participants with achondroplasia were assessed for eligibility. Fifteen controls and five participants with achondroplasia were excluded for not meeting inclusion and/or matching criteria. The final analytical sample comprised 68 controls and 33 participants with achondroplasia who completed anthropometric and body composition assessments.

Children and adolescents with a confirmed clinical and radiological diagnosis of achondroplasia and age and sex-matched controls were recruited. Achondroplasia was diagnosed on the basis of characteristic clinical and radiological findings and confirmed by identification of a pathogenic FGFR3 mutation when available (2). Inclusion criteria for both groups were age between 6 and 18 years and absence of chronic diseases or medication use known to affect growth or body composition (Figure 1). Controls were recruited either for an annual routine health checkup (healthy children without long-term diseases) or consulting for excess weight. Inclusion criteria were absence of chronic disease, skeletal dysplasia, endocrine disorders, or conditions known to affect growth or body composition. Participants with acute illness at the time of evaluation were excluded. Participants with other forms of skeletal dysplasia or endocrine disorders were excluded. Participants were classified into three developmental groups according to standard paediatric categories: middle childhood (6–11 years), early adolescence (12–14 years), and middle adolescence (15–18 years). A cross-sectional analysis was performed with 101 (53 females/48 males) White children and adolescents, recruited between July 2023 and July 2025 at the Departments of Endocrinology and Nutrition from the Virgen de la Victoria Hospital, Malaga, and from the Clínica Universidad de Navarra, Pamplona, both located in Spain.

### Body composition

Anthropometric and body composition assessments were performed during a single study visit by following standardized procedures. All anthropometric and body composition measurements were performed by the same trained investigator in each centre to minimize inter-observer variability. Height was measured to the nearest 0.1 cm using a Holtain stadiometer (Holtain, Crymych, UK). Body weight was measured using the calibrated electronic scale integrated into the ADP system (BOD POD^®^, COSMED, Albano Laziale, Italy), with participants wearing tight-fitting clothing and a swim cap, according to the manufacturer’s recommendations. BMI was calculated as weight (kg) divided by height squared (m^2^). ADP calculates body density from measured body mass and body volume. ADP was selected because it is rapid, safe, well tolerated by children, and does not involve exposure to ionizing radiation. Since the use of ADP requires patient cooperation and may be challenging in younger children, only children aged 6 years and older were included in the study. Thoracic gas volume was measured by the huffing methodology following the manufacturer’s recommendations. BF% was derived from body density using the Siri equation. Fat mass (FM) and FFM were subsequently calculated from body weight and BF%. Although ADP provides accurate estimates of whole-body composition and has been extensively validated in pediatric populations (15–17), it does not provide information on regional fat distribution, which represents one of its main limitations. FM index (FMI) and FFM index (FFMI) were calculated as FM (kg) or FFM (kg) divided by height squared (m^2^), respectively: FMI = FM (kg)/height^2^ (m^2^); FFMI = FFM (kg)/height^2^ (m^2^) (18, 19).

### Statistical analysis

The required sample size was estimated *a priori* based on detecting a large effect size (Cohen’s d = 0.80) in group comparisons of FM and FFM, with a power of 90% and an alpha level of 0.05 and an allocation ratio of 1/2. This calculation indicated that a minimum of 25/51 participants was needed, and our final sample met or exceeded this requirement. Data are presented as mean ± standard deviation (SD). The normal distribution of the continuous variables was assessed using the Kolmogorov–Smirnov test. Differences between groups were analyzed by two-tailed unpaired Student’s *t* tests or two-way ANOVA. Homogeneity of variances was assessed using Levene’s test prior to conducting the two-way ANOVA. No observations were excluded as outliers. Correlations were performed using Pearson’s correlation analysis. The calculations were performed by SPSS version 23 (Chicago, IL, United States) and GraphPad Prism 8 (La Jolla, CA, United States). *p* values lower than 0.05 were considered statistically significant.

### Results

A total of 101 children and adolescents were included in the analysis, comprising 33 participants with achondroplasia (19 boys and 14 girls) and 68 control participants (29 boys and 39 girls). The anthropometric and body composition characteristics of the study population, stratified by sex and group, are summarized in Table 1. The age distribution was comparable across groups, with mean ages ranging from 9.9 to 10.9 years, indicating that potential age-related differences in growth and development were minimized.

**Table 1:** Characteristics of children and adolescents included in the analysis segregated by sex.

	Boys		Girls	
	Control	Achondroplasia	Control	Achondroplasia
n	29	19	39	14
Age, y	10.9 ± 2.9	10.3 ± 2.9	9.9 ± 3.1	10.9 ± 3.0
Height, m	1.46 ± 0.11	1.14 ± 0.14***	1.41 ± 0.14	1.10 ± 0.14***
Weight, kg	48.0 ± 13.9	32.2 ± 10.7***	44.6 ± 16.9	30.2 ± 10.3**
BMI, kg/m^2^	22.3 ± 4.9	24.6 ± 6.6	21.9 ± 4.4	24.7 ± 6.3
Body fat, %	30.2 ± 7.4	27.7 ± 12.6	33.4 ± 7.1	28.3 ± 6.6*
Body fat, kg	15.2 ± 8.0	9.8 ± 6.6*	15.5 ± 9.6	8.9 ± 4.5*
Fat-free mass, %	69.8 ± 7.4	72.3 ± 12.6	66.6 ± 7.1	71.7 ± 6.6*
Fat-free mass, kg	32.8 ± 7.1	22.4 ± 5.9***	29.1 ± 8.3	21.3 ± 6.4**
FMI, kg/m^2^	7.0 ± 3.2	7.4 ± 5.0	7.6 ± 3.2	7.3 ± 3.7
FFMI, kg/m^2^	15.3 ± 2.0	17.2 ± 2.7**	14.4 ± 1.6	17.4 ± 3.0**

Data are mean ± SD.

BMI, body mass index; FMI, fat mass index; FFMI, fat-free mass index.

*p < 0.05, **p < 0.01, and ***p < 0.001, vs control.

### Anthropometric characteristics

**Figure 2:**
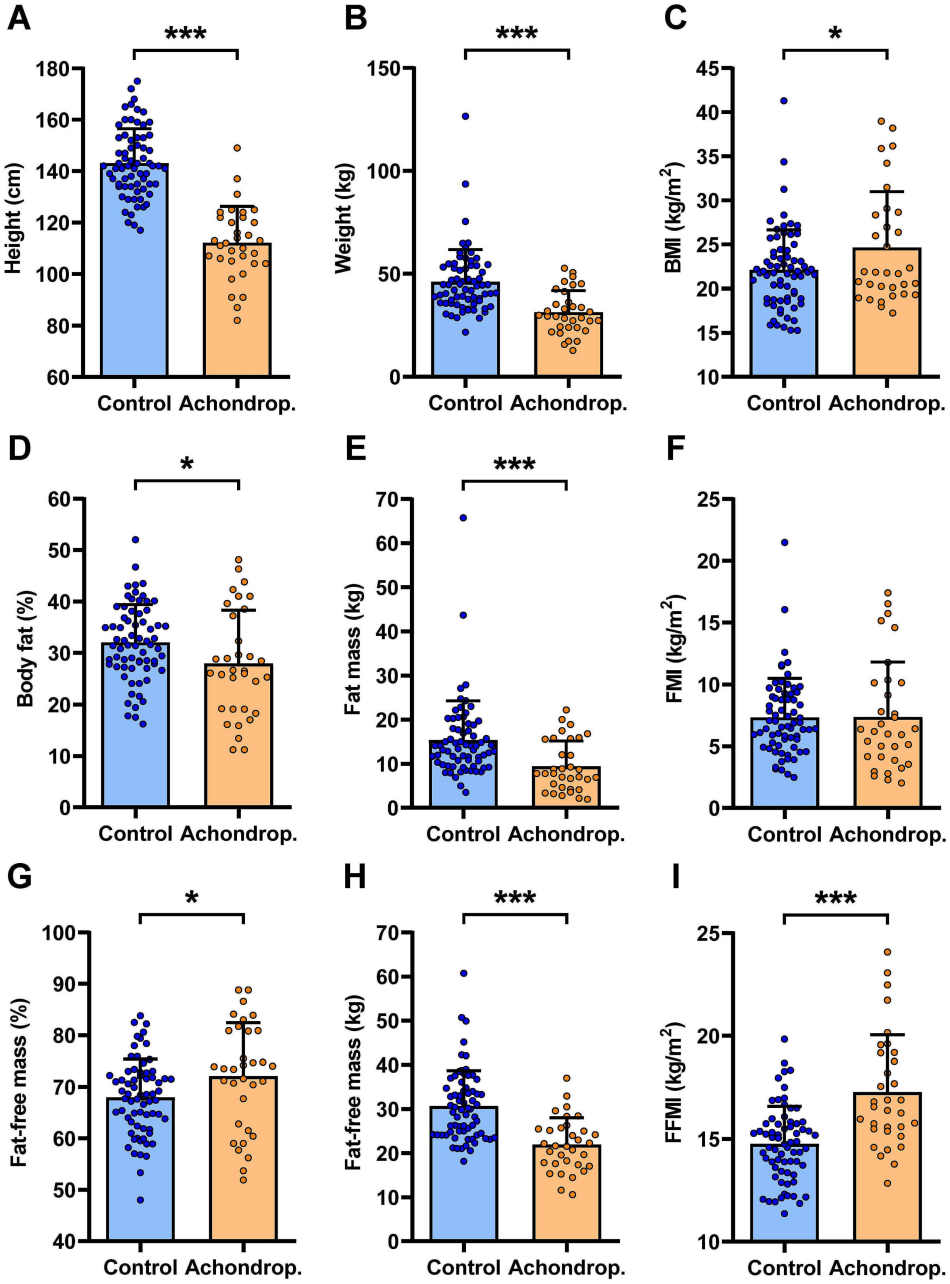
Anthropometric and body composition differences between participants with achondroplasia (*n* = 33) and controls (*n* = 68). Height **(A)**, weight **(B)**, BMI **(C)**, body fat percentage **(D)**, fat mass **(E)**, fat mass index (FMI) **(F)**, fat-free mass percentage **(G)**, fat-free mass **(H)**, and fat-free mass index (FFMI) **(I)** are shown for controls and participants with achondroplasia. Bars represent mean ± SD. Group comparisons were performed using unpaired Student’s *t* tests. *p < 0.05 and ***p < 0.001.

Marked differences in linear growth were observed between children with achondroplasia and their peers without the condition. Mean height was approximately 22% lower among participants with achondroplasia in both sexes. Boys with achondroplasia had an average height of 1.14 ± 0.14 m compared with 1.46 ± 0.11 m in control boys (*p* < 0.001), while girls with achondroplasia measured 1.10 ± 0.14 m compared with 1.41 ± 0.14 m in controls (*p* < 0.001) (Figure 2). Similarly, body weight was significantly lower in the group with achondroplasia (boys: 32.2 ± 10.7 kg vs. 48.0 ± 13.9 kg; girls: 30.2 ± 10.3 kg vs. 44.6 ± 16.9 kg; all *p* < 0.01). When boys and girls were analyzed together, BMI was significantly higher in participants with achondroplasia compared with controls (*p* < 0.05). However, when stratified by sex, BMI differences were not statistically significant, suggesting that BMI alone may not adequately reflect body composition differences in this population.

### Body composition

**Figure 3:**
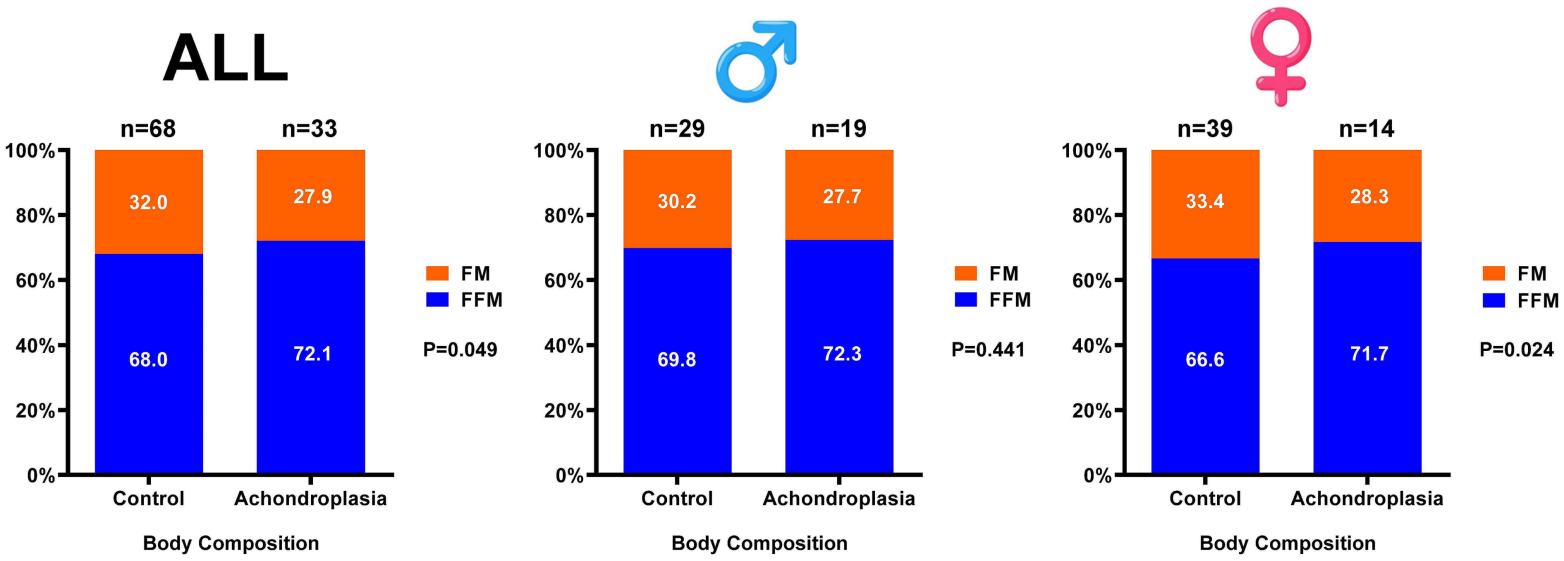
Relative fat mass and fat-free mass distribution in participants with achondroplasia and controls. Stacked bar graphs depict the proportion of fat mass (FM) and fat-free mass (FFM) for the entire cohort (left), males (center), and females (right). Sample sizes for each subgroup are indicated. Differences between groups were assessed using unpaired Student’s *t* tests, with *p*-values shown for each comparison. Since the proportions of FM and FFM are complementary (both values add up to 100) the *p* values for both variables are the same in each comparison.

Distinct body composition patterns were identified between groups (Table 1 and Figures 2, 3). Absolute FFM was significantly lower in children and adolescents with achondroplasia (boys: 22.4 ± 5.9 kg vs. 32.8 ± 7.1 kg; girls: 21.3 ± 6.4 kg vs. 29.1 ± 8.3 kg; *p* < 0.01). In contrast, the FFMI was significantly higher in the group with achondroplasia (boys: 17.2 ± 2.7 vs. 15.3 ± 2.0; girls: 17.4 ± 3.0 vs. 14.4 ± 1.6; *p* < 0.01). This apparent paradox reflects the shorter stature of participants with achondroplasia, which results in a higher ratio of lean mass relative to height squared.

FM and BF% were also reduced in the group with achondroplasia compared with the control group. Boys with achondroplasia exhibited lower FM values (9.8 ± 6.6 kg vs. 15.2 ± 8.0 kg; *p* < 0.05), and a similar difference was observed among girls (8.9 ± 4.5 kg vs. 15.5 ± 9.6 kg; *p* < 0.05). BF% followed the same trend, with lower values in the group with achondroplasia (boys: 27.7 ± 12.6% vs. 30.2 ± 7.4%; girls: 28.3 ± 6.6% vs. 33.4 ± 7.1%) reaching significant differences only in girls (*p* < 0.05). However, the FMI did not differ significantly between groups, indicating that proportional fatness adjusted for height squared may be comparable between children with and without achondroplasia.

### Age-related differences

**Figure 4:**
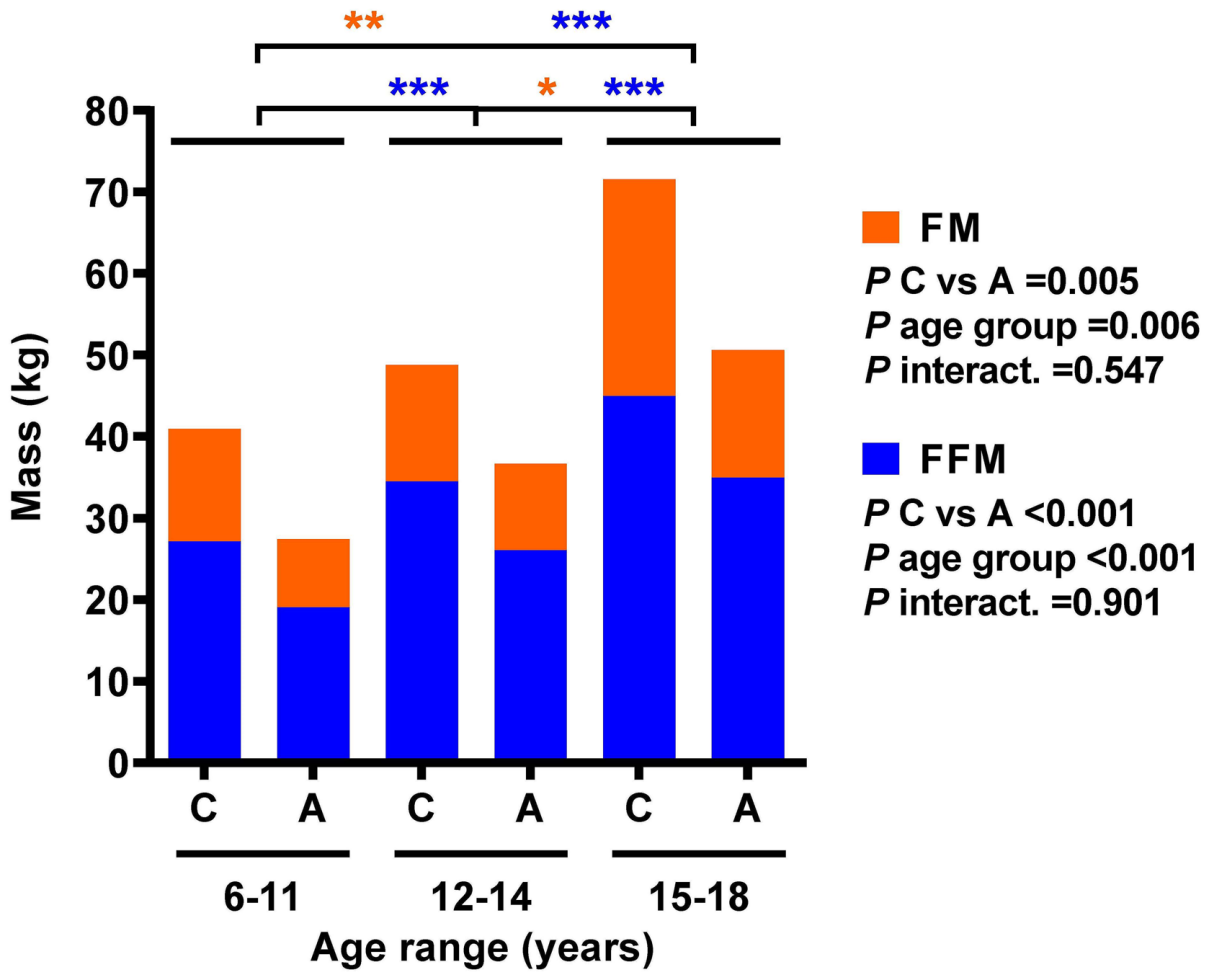
Fat mass and fat-free mass across age groups in participants with achondroplasia and controls. Stacked bars represent mean fat mass (FM) and fat-free mass (FFM) for children and adolescents aged 6–11, 12–14, and 15–18 years. Analyses compare controls (C) and participants with achondroplasia (A). Main effects for group and age, and group × age interactions, were evaluated using two-way ANOVA (*p*-values shown on the right).

**Figure 5:**
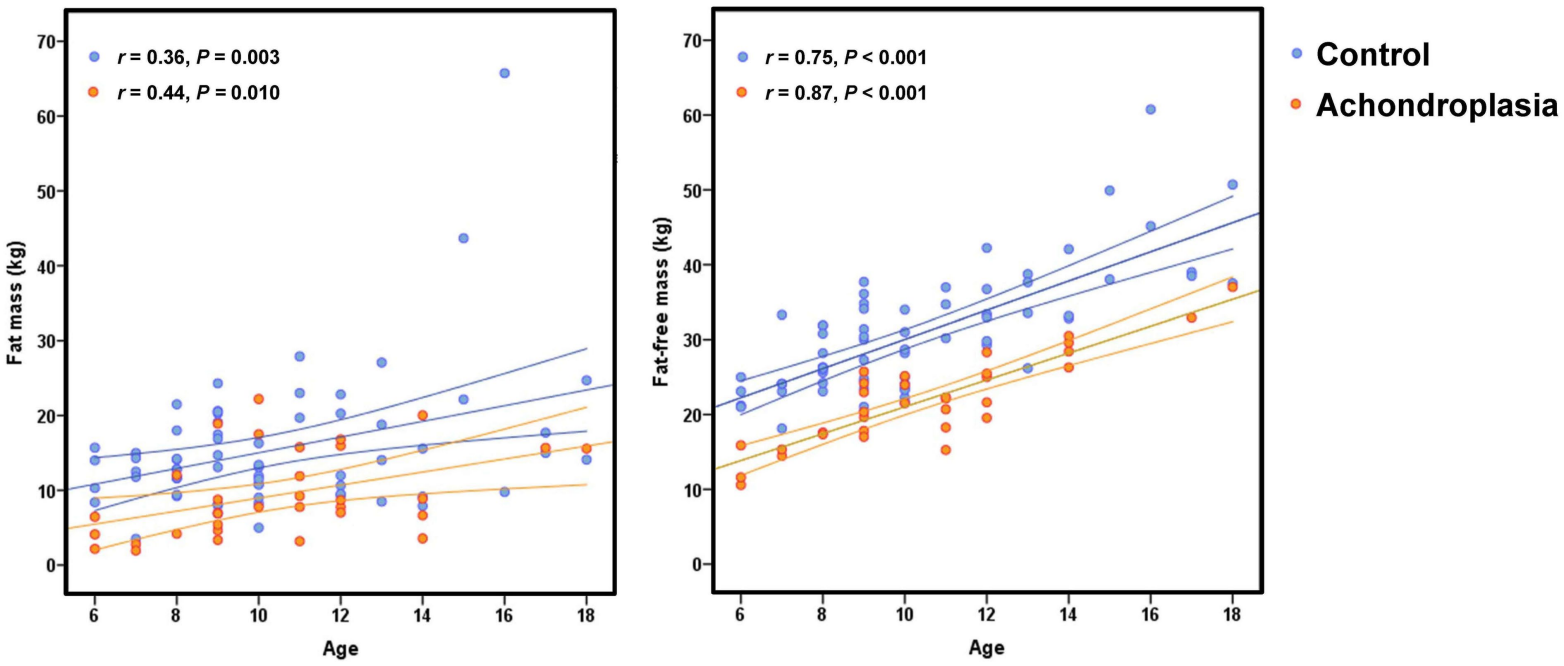
Associations of age with fat mass and fat-free mass in participants with achondroplasia (*n* = 33) and controls (*n* = 68). Scatterplots with regression lines show the relationships between age and fat mass (left) and fat-free mass (right). Data are presented separately for controls and participants with achondroplasia. Pearson correlation coefficients (*r*) and corresponding *p*-values are shown in each panel.

To investigate differences in body composition across developmental stages, participants were categorized into three predefined age groups (6–11, 12–14, and 15–18 years). As shown in Figure 4, older age groups exhibited higher absolute FM and FFM values in both the control and achondroplasia groups. The two-way ANOVA demonstrated significant main effects of group (control *vs*. achondroplasia) and age category for both FM (*p* = 0.005 and *p* = 0.006, respectively) and FFM (both *p* < 0.001), with no significant group-by-age interaction (all *p* > 0.5). *Post hoc* comparisons revealed significantly higher FM in participants aged 15–18 years compared with those aged 6–11 years (*p* = 0.004) and 12–14 years (*p* = 0.030), whereas the difference between the 6–11 and 12–14 year groups was not statistically significant. For FFM, all pairwise comparisons between age groups were significant (*p* < 0.001 for all). These findings indicate that although body composition differed between participants with achondroplasia and controls, the pattern of differences across age groups was similar in both groups, as reflected by the absence of a significant group-by-age interaction. Correlation analyses were performed to examine the relationship between age and body composition. As illustrated in Figure 5, age was strongly correlated with FFM both in participants with achondroplasia (*r* = 0.87, *p* < 0.001) and controls (*r* = 0.75, *p* < 0.001). In contrast, age showed only moderate associations with FM both in participants with achondroplasia (*r* = 0.44, *p* = 0.010) and controls (*r* = 0.36, *p* = 0.003).

Overall, these findings demonstrate that while children and adolescents with achondroplasia have markedly reduced stature and total tissue mass, their relative indices of body composition (FMI and FFMI) reveal distinct proportional adaptations.

## Discussion

Our study provides detailed anthropometric and body composition data in children and adolescents with achondroplasia compared with age-matched controls. Our main findings indicate that participants with achondroplasia present markedly reduced stature and absolute tissue masses (both FFM and FM in kg) but exhibit higher FFMI and comparable FMI relative to controls. When data were analyzed by sex, BMI differences were not significant; however, when boys and girls were combined, BMI was significantly higher in the group with achondroplasia.

Body composition was measured by ADP using the BOD POD system, which offers high precision and accuracy in estimating body volume and density. Fields et al. confirmed that ADP shows excellent agreement with four-compartment models in children and adults, with coefficients of variation below 3.5% (15). Compared with other field techniques such as bioelectrical impedance or skinfolds, ADP is less operator-dependent and more reproducible (20, 21), making it suitable for the evaluation of children with skeletal dysplasias where body geometry deviates from reference assumptions.

The present data confirm the characteristic disproportionate short stature of achondroplasia, with average height approximately 22% lower than in control peers. Absolute lean and fat masses were significantly reduced in both sexes, whereas FFMI was higher and FMI similar between groups. The absence of significant differences in FMI between participants with achondroplasia and controls despite marked differences in height, body weight, and absolute body composition variables is one of the most relevant findings of the present study. This observation suggests that the higher BMI frequently reported in achondroplasia may largely reflect reduced stature rather than excess adiposity. Because the FMI adjusts fat mass for height, it may provide a more appropriate index of adiposity in this population than the BMI alone. These results mirror previous reports showing that, although people with achondroplasia have reduced total muscle and bone mass, their height-adjusted indices may appear adequate or elevated due to the mathematical influence of short stature (13, 22). The natural-history CLARITY cohort, which provided detailed growth charts for children with achondroplasia, also demonstrated the limitations of BMI and weight-for-height in this population (22). To our knowledge, this is the first study to characterize detailed body composition patterns across childhood and adolescence in achondroplasia using ADP, providing age-specific insights that were previously unavailable. A key novelty of this work is the comprehensive, age-stratified assessment of fat and lean mass in achondroplasia, which reveals developmental trajectories that have not been described in the literature. The consistent patterns across age groups suggest that the unique body composition profile of achondroplasia is established early and persists throughout growth.

The apparent paradox of higher FFMI but lower absolute lean mass can be explained by the disproportion between height and tissue mass. Because FFMI divides lean mass by height squared, lower height increases FFMI even when lean mass is reduced. This artifact has been described in other short-stature conditions and underscores the need for caution when applying BMI or height-based indices to populations with altered body proportions (23). Importantly, our data confirm that BMI alone cannot accurately reflect nutritional or metabolic status in achondroplasia.

When both sexes were combined, BMI was significantly higher in participants with achondroplasia, consistent with previous observations that BMI tends to overestimate adiposity in short individuals (6, 10). However, sex-stratified analyses did not show significant differences, suggesting that the elevated combined BMI may largely reflect differences in body proportion rather than increased adiposity. BMI combines mass and stature, and its use in achondroplasia is particularly problematic because of reduced limb length and increased trunk proportion. Recent paediatric studies have shown that the BMI can misclassify up to 30% of children when compared with direct measures of FM (8, 24). Thus, indices that independently quantify fat (like the FMI) and lean mass (like the FFMI), or direct assessment methods such as ADP or DXA, provide a more accurate evaluation of body composition in children with achondroplasia. As expected, girls exhibited higher body fat percentages than boys in both groups, reflecting well-established sex-specific differences in body composition that emerge during childhood and become more pronounced during puberty (25).

From a clinical perspective, our findings have several implications. First, routine monitoring of growth and nutritional status in children and adolescents with achondroplasia should include body composition parameters, particularly FFMI and FMI, rather than BMI alone. The most recent clinical practice guidelines for achondroplasia emphasize that BMI must be interpreted with caution and that body composition assessment should be incorporated into follow-up protocols (6). Second, the lower absolute lean mass observed here could have metabolic and functional consequences. Because skeletal muscle is the primary determinant of resting energy expenditure (REE), the lower FFM could theoretically influence energy requirements and influence glucose and lipid metabolism (26). Although data on metabolic health in children with achondroplasia remain limited (27), studies in adults suggest that central adiposity and cardiometabolic comorbidities such as insulin resistance and dyslipidaemia may be more frequent (28). Whether these alterations originate during childhood and are linked to reduced lean mass or altered fat distribution remains to be clarified.

Strengths of the present study include the use of a direct and precise body composition method (ADP) and stratified analyses by sex and age, which permitted assessment of parallel developmental trajectories in fat and lean mass. The observation that differences in both compartments were comparable across age groups, following similar slopes in achondroplasia and control groups suggests that the relative differences are established early and maintained through adolescence.

### Study limitations

Several limitations must also be considered. The sample size, particularly within sex-age strata, was modest and may have limited statistical power for detecting small sex-specific effects. Although ADP has excellent precision in children, its accuracy may be reduced in populations with atypical body geometry or respiratory volume assumptions (29). Finally, the cross-sectional design prevents inference about causality or growth trajectories; longitudinal studies are needed to determine whether the observed body composition patterns persist or change across developmental stages.

### Clinical implications

Future research should focus on prospective cohorts using repeated ADP or DXA measures tracking the evolution of fat and lean compartments and examine associations with metabolic outcomes such as insulin sensitivity, lipid profile, and REE. Development of achondroplasia-specific percentile charts for FFMI, FMI, and possibly waist-to-height ratios would improve clinical monitoring and avoid misclassification of nutritional status. Finally, tailored nutritional and physical-activity programmes in children and adolescents with achondroplasia designed to preserve or increase lean mass without promoting excess fat accumulation should be evaluated for their effects on metabolic health and physical function.

## Conclusion

Children and adolescents with achondroplasia exhibit markedly reduced absolute body mass but maintain a proportional distribution of fat and lean tissue relative to height. Elevated FFMI and normal FMI values indicate that the BMI overestimates adiposity in this population. The use of height-adjusted indices and direct composition assessment will be essential to accurately monitor growth, identify nutritional risks, and guide interventions aimed at improving metabolic health and functional capacity in people with achondroplasia.

## Data Availability

The datasets presented in this article are not readily available due to ethical restrictions and the need to protect participant confidentiality. Access to anonymized data may be considered upon reasonable request and subject to approval by the Institutional Review Board of Virgen de la Victoria Hospital and Clínica Universidad de Navarra. Requests to access the datasets should be directed to the corresponding author, jagomez@unav.es.
